# The good pharmacy practice on Einstein Program at Paraisópolis Community

**DOI:** 10.1590/S1679-45082016GS3751

**Published:** 2016

**Authors:** Lara Tânia de Assumpção Domingues Gonçalves de Oliveira, Camila Pontes da Silva, Maria das Vitorias Guedes, Ana Célia de Oliveira Sousa, Flávio Sarno

**Affiliations:** 1 Sociedade Beneficente Israelita Brasileira Albert Einstein, São Paulo, SP, Brazil; Hospital Israelita Albert Einstein, São Paulo, SP, Brazil.; Hospital Israelita Albert Einstein, Hospital Israelita Albert Einstein, São Paulo, SP, Brazil

**Keywords:** Pharmacies, Ambulatory care facilities, Child health services, Comprehensive health care

## Abstract

**Objectives:**

To describe indicators and processes developed and implemented for pharmaceutical assistance at the Einstein Program at Paraisópolis Community pharmacy.

**Methods:**

This was a descriptive study of retrospective data from January 2012 to December 2015. Data were obtained from spreadsheets developed for monitoring the productivity and care quality provided at the pharmacy. The evaluated variables were pharmaceutical assistance to prescription, pharmaceutical intervention, orientation (standard and pharmaceutical) and pharmaceutical orientation rate.

**Results:**

The pharmacy assisted, on average, 2,308 prescriptions monthly, dispensing 4,871 items, including medications, materials and food supplements. Since March 2015, virtually, the pharmacist analyzed all prescriptions, prior to dispensing. In the analyzed period, there was an increase in monthly pharmaceutical interventions from 7 to 32 on average, and, although there was a decrease in the number of standard orientation, the pharmaceutical orientation had an increase, causing a rise of pharmaceutical orientation rate from 4 to 11%.

**Conclusion:**

The processes developed and implemented at the program pharmacy sought to follow the good pharmacy practice, and help patients to make the best use of their medications.

## INTRODUCTION

The International Pharmaceutical Federation and World Health Organization define good pharmacy practice (GPP) as practices that meet personal needs of those using pharmacy services by offering appropriate evidence based care.^(1)^

In Brazil, pharmaceutical assistance was defined as a pharmaceutical practice model that involves attitudes, ethical values, behaviors, skills, appointments and co-responsibility to prevent diseases, promote and recovery health in an integrated manner as part of the health care process, highlighting, among other, the requirement that the institution fully adopt the GPP.^(2)^ Technical regulations of GPP was approved by the Federal Council of Pharmacy by resolution 357 and 416.^(3)^

In 2004, the Brazilian Ministry of Health approved the national policies for pharmaceutical assistance as part of the national policies of health involving a set of actions to promote, protect and recovery health, and assure principles of universality, integrality and equity, assuming the medication as an essential inputs and guarantee access to and rational use.^(4)^

Therefore, pharmaceutical assistance and care, using GPP as guidelines, are important in maintenance of principles that regulate Brazilian Health System.

The Einstein Program at Paraisópolis Community (PECP, *Programa Einstein na Comunidade de Paraisópolis*) was inspired in the actions of the *Sociedade Beneficente Israelita Brasileira Albert Einstein* and, currently, offers broad multidisciplinary and specialized pediatric care and develops social-educational activities for the community.^(5)^ The PECP pharmacy seeks to contribute with the adequate use of drug therapy for patients focusing on GPP and ensuring access to medication and information related with treatment.

## OBJECTIVE

To describe indicators and processes developed and implemented for pharmaceutical assistance in the pharmacy of the Einstein Program at Paraisópolis Community.

## METHODS

This was a descriptive, cross-sectional study including retroactive data from January 2012 to December 2015 at pharmacy of PECP. The pharmacy is located in the outpatient unit at PECP and it provides pharmaceutical care, prescribe medications, materials and food suppliers upon requested in the multidisciplinary care at PECP, as well as in urgencies and emergencies at other health units of the region.

Data were obtained from spreadsheets specifically developed for use of professionals (two pharmacists and two pharmacy assistants) in the follow-up of productivity and quality of care provided in the pharmacy.

Variables were:

– Pharmaceutical assistance for prescription: number of prescriptions analyzed by pharmacist. Before to dispense medications and food supplements for the first time, pharmacists should check patients' identification, legibility, pertinence, indications, interactions, compatibility, allergies, dose, frequency and time of treatment of prescribed items.
– Pharmaceutical intervention: number of situations in which the pharmacist detect the possibility of drug-related problems (DRPs), defined as avoidable events involving drug therapy that can potentially interfere in the desired treatment.^(6)^ The pharmacist should revert or avoid such events.
– Guidance: a number of explanations by pharmacists during assistance and provision of medications, materials or food supplements. They are divided into:
  – Standard guidance: during dispensing process, professionals in the pharmacy should read the prescription to the responsible for the patient to make the understanding easy about the posology and time of treatment, conservation, expiration date, and adequate disposal of items. At this time, the patient or responsible is stimulated to reported side effects and, if any has occurred. The number of standard guidance represents the number of prescriptions made, excluded and those that required guidance from the pharmacy.
  – Pharmaceutical guidance: situations considered relevant, pharmacy provides, in addition to information provided in standard guidance, the basic information on indications, side effects, medication-medication and medication-food interactions, importance of adherence to treatment, administration techniques, hygiene, risk of fall, allergies and intolerances and medication reconciliation. Situations that involve pharmacy guidance are: beginning treatment with inhalation medication, use of medications that side effects can be severe (systemic corticoids, non-steroids anti-inflammatories for younger than 12 years, immunosuppressant drugs, anticoagulants, digitalis and antiarrhythmic agents), beginning treatment with medications that act on central nervous system, beginning treatment with injectable hormones, beginning treatment or prophylaxis for tuberculosis, treatment with systemic antimicrobial agents, complex treatments (which require actions at multiple times or specific administration techniques), beginning treatment for anemia, when observed low adherence to the previously implemented treatment, when patient or responsible for the patient has difficult in understanding, when requested by the prescriber, and when requested by the patient.
  – Pharmaceutical guidance rates: number of pharmaceutical guidance done concerning total of guidance (standard guidance summed to pharmaceutical guidance), expressed in percentages.

Variables were organized in spreadsheets by year of occurrence and presented in absolute number and frequency of occurrence.

The study was approved by the Ethical and Research Committee of the *Hospital Israelita Albert Einstein*, number 1.335.625 of November 24, 2015 and number 1.353.819 of December 7, 2015, CAAE: 50844715.5.0000.0071. The consent term was waived by the institution for the study.

## RESULTS

Between 2012 and 2015, pharmacy of PECP assisted, on average, 2,308 prescriptions monthly with 4,871 items dispensed, including medications, materials and food supplements.

The process of pharmaceutical assistance to prescriptions existed since 2006 and, from July 2012, the flow of assistance had changed in such a way that, before dispensing, the pharmaceutical professional can analyze each prescription. Since that time, almost all prescriptions were analyzed by the pharmacist (Table 1).

**Table 1:** Prescriptions analyzed by the pharmacist concerning the total of prescriptions given at Einstein program in Paraisópolis Community, according to year and month of occurrence

Month	Year			
	2012 (%)	2013 (%)	2014 (%)	2015 (%)
January	37.9	90.2	90.5	98.2
February	43.2	99.0	98.7	100
March	48.4	99.4	100	100
April	44.1	100	98.7	100
May	44.7	99.1	100	100
June	61.9	100	100	100
July	100	100	100	100
August	98.6	100	100	100
September	98.5	99.2	100	100
October	99.8	100	99.8	100
November	98.2	100	99.6	100
December	98.9	100	98.9	100
Mean in the year	72.9	98.9	98.9	99.9

The assessment of possible occurrence of DRP was introduced in the pharmacy of PECP in March 2012 and, until 2014, a total of seven to eight pharmaceutical interventions were carried out annually. In February 2015, there was including of record of pharmacist performance in situations requested by user, such as loss of prescription, spontaneous report of inefficiency of treatment and lack of understanding information during care prescribing. From that time, the number pharmaceutical interventions increased to mean of 32 interventions yearly (Table 2).

**Table 2:** Pharmaceutical interventions, according to year and month of occurrence

Month	Year			
	2012	2013	2014	2015
January	0	7	4	0
February	0	6	5	34
March	0	11	4	19
April	1	10	5	19
May	13	7	7	13
June	0	4	2	36
July	18	5	13	42
August	17	12	7	47
September	16	10	11	48
October	13	12	10	47
November	5	6	13	51
December	5	8	2	33
Mean in the year	7.3	8.2	6.9	32.4

**Figure 1:**
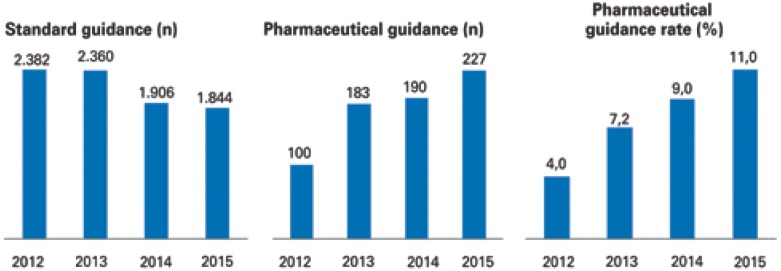
Mean in the month of standard guidance and pharmaceutical guidance and rate of pharmaceutical guidance according to year of occurrence

Between 2012 and 2015, there was reduction in number of standard guidance done. However, the number of pharmaceutical guidance increased 2.3 times in the same period. Therefore, there was an increase in pharmaceutical guidance rate of 4% in 2012 for 11% in 2015 (Figure 1).

**Figure 2:**
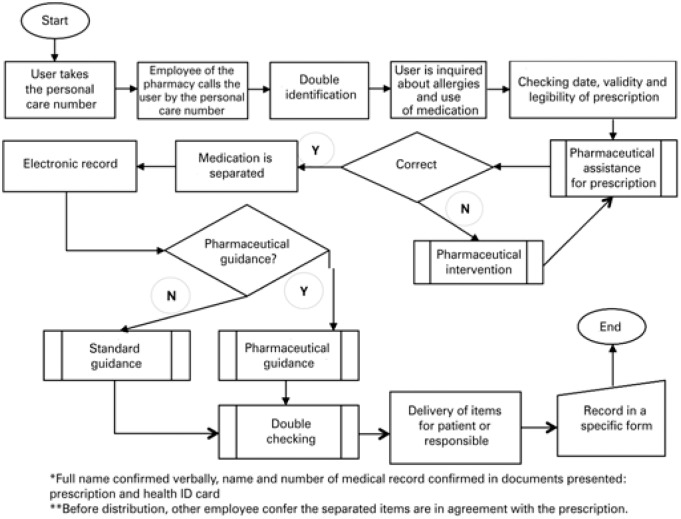
Flow of assistance of user in pharmacy of Einstein Program at Paraisópolis Community

Flow of assistance of user in pharmacy of PECP showing all processes involved in disposing prescribed items (Figure 2).

## DISCUSSION

The GPP are organized in four main roles of the pharmacists: prepare, obtain, store, protect, distribute, administrate, dispose and provide medical products; provide efficient management of drug therapy, keep and improve professional performance, and contribute to improve the effectiveness of health care system and public health system.^(1)^ Our study had indicators and processes developed and implemented in the pharmacy of PECP to dispense medications, materials and supplements, and provide efficient pharmaceutical assistance. Changes involved the flow of patients and prescriptions, without changes involving costs.

Pharmaceutical assistance and pharmaceutical care are different concepts: the first involves a broad set of actions with multidisciplinary characteristics, and the latter is related to specific activities of pharmacist that aims rational pharmacotherapy.^(2)^ However, we observed that only 2.5% of professionals perform pharmaceutical care recommend by Brazilian guidelines.^(7)^ Difficulties presented to implement pharmaceutical care involves time, lack of evidence of cost-effectiveness of process and formal knowledge of pharmacists for development of this activity.^(8–10)^ In the pharmacy of PECP, the development of processes involving DRP occurred gradually, evaluating daily indicators, identifying and correcting difficulties quickly, reducing impacts in the flow of patients.

There are few studies in Brazil evaluating pharmaceutical assistance and care. Good results are found in stages of storing, distribution and transportation, however, prescription was evidenced as more critical stage of the process.^(11)^ Fails were detected also in reference to other health professionals such and communication with physicians.^(12)^ Lack of adequate structure for private care of patients was also observed.^(13)^ These studies used a number of instruments of assessment, but they were based in the model structure, process and result. In our study, the assessment of pharmaceutical assistance was performed by using related indicators with professional performance in the pharmacy in order to avoid DRP, which is a barrier for comparison with these studies.

Medication errors can occur in any phase of the process of use of medication.^(14)^ The prescription was considered more critical, and the population at more risk to errors were elderlies and those younger than 18 years old.^(15)^ In addition, in postcommercialization phase of the medication no established side effects can occur.^(16)^ Therefore, it is important to monitor DRP routinely in all phase of the use of medications.

Results showed that pharmaceutical assistance increases adherence to drug therapy, resolves the majority of pharmacotherapy problems and improves control of clinical parameters of the disease. Although no study directly evaluated quality of life and economic impact of interventions,^(17)^ evidences exist concerning satisfaction degree of users of services that provide pharmaceutical care.^(18)^ Despite the different intervention strategies available, it was impossible possible to determine one that can improve all results within all populations, diseases and local of implementation.^(19)^ Our study did not evaluate health outcomes, however, we evaluated indicators that can be seen as proximal variables (proxy) of outcomes, *i.e*., better pharmaceutical guidance and intervention, *i.e.*, better pharmacological guidance and intervention that can be translated as reduction of DPR and bring positive impact in patients' lives.

Interventions developed in pharmacy practice must be cost-effective to be incorporated in the service without compromise sustainability.^(20)^ Studies that evaluated this issue estimated that return on investment based on time spending by pharmacists, identifying DRP and cost of avoidable medical services ranged from 1.25 – 1.5.^(21,22)^ Therefore, interventions can be adapted to local reality, based on previous analysis of service in order to identify types and where DRP are occurring in order to provide efficient interventions.

Dispensing of medication involves a number of actions,^(23)^ and the pharmacist has little time and not always has all information needed for complete assessment of medicines. In addition, prescription cannot present all criteria needed for correct and safety use of medications.^(24)^ Therefore, dispensing should be rethink, and all essential processes should be presented. In addition, pharmaceutical practice service reality should be considered.^(25)^ The pharmacy of PECP defined a flowchart for dispensing which enable professionals of the department to evaluate each prescription adequately without compromise the flow of patients.

The seeking for a prescription that promotes safety, efficacy and efficiency in the use of medication is ideal to all caregivers involved in patient's care. Currently, electronic prescription^(26)^ is considered easy to receive and process, and it often fulfill the requirements of dispensing pharmacy.^(27)^ However, in addition to prescription, the pharmaceutical practice must improve health care quality more broadly, taking the opportunities such as to act in medication reconciliation, adherence to medication and help the patient with self-management of his/her medication.^(28)^

## CONCLUSION

Procedures based on good pharmacy practice can be elaborated and implemented. Processes developed and established in the pharmacy of Einstein Program at Paraisópolis Community can be a model and a stimulus to other professionals and services seeking better use of medications.
